# Use of Autologous Human mesenchymal Stromal Cell/Fibrin Clot Constructs in Upper Limb Non-Unions: Long-Term Assessment

**DOI:** 10.1371/journal.pone.0073893

**Published:** 2013-08-30

**Authors:** Stefano Giannotti, Luisa Trombi, Vanna Bottai, Marco Ghilardi, Delfo D'Alessandro, Serena Danti, Giacomo Dell'Osso, Giulio Guido, Mario Petrini

**Affiliations:** 1 Dept. of Translational Research and New Technology in Medicine and Surgery, University of Pisa, Pisa, Italy; 2 Dept. of Clinical and Experimental Medicine, Hematology Division, University of Pisa, Pisa, Italy; 3 Dept. of Surgical, Medical, Molecular Pathology and Emergency, University of Pisa, Pisa, Italy; Universidade do Porto, Portugal

## Abstract

**Background:**

Tissue engineering appears to be an attractive alternative to the traditional approach in the treatment of fracture non-unions. Mesenchymal stromal cells (MSCs) are considered an appealing cell source for clinical intervention. However, *ex vivo* cell expansion and differentiation towards the osteogenic lineage, together with the design of a suitable scaffold have yet to be optimized. Major concerns exist about the safety of MSC-based therapies, including possible abnormal overgrowth and potential cancer evolution.

**Aims:**

We examined the long-term efficacy and safety of *ex vivo* expanded bone marrow MSCs, embedded in autologous fibrin clots, for the healing of atrophic pseudarthrosis of the upper limb. Our research work relied on three main issues: use of an entirely autologous context (cells, serum for *ex vivo* cell culture, scaffold components), reduced *ex vivo* cell expansion, and short-term MSC osteoinduction before implantation.

**Methods and Findings:**

Bone marrow MSCs isolated from 8 patients were expanded *ex vivo* until passage 1 and short-term osteo-differentiated in autologous-based culture conditions. Tissue-engineered constructs designed to embed MSCs in autologous fibrin clots were locally implanted with bone grafts, calibrating their number on the extension of bone damage. Radiographic healing was evaluated with short- and long-term follow-ups (range averages: 6.7 and 76.0 months, respectively). All patients recovered limb function, with no evidence of tissue overgrowth or tumor formation.

**Conclusions:**

Our study indicates that highly autologous treatment can be effective and safe in the long-term healing of bone non-unions. This tissue engineering approach resulted in successful clinical and functional outcomes for all patients.

## Introduction

Pseudarthrosis (or non-union) is a frequent complication occurring after surgical treatment of fractures, and results in a major challenge for orthopedic surgery [1], [2]. Osteotomy followed by bone distraction (Ilizarov method) and auto/allo-grafting represents the most common therapeutic approach [3]. However, the current techniques available do not guarantee complete osteointegration and recovery of natural bone mechanical properties.

Various strategies, including tissue engineering, have been used to promote osteogenesis. Embedding viable cells within biological or artificial matrices/scaffolds appears to be extremely promising since it allows osteocompetent cells to generate new bone tissue, thus contributing to tissue integration and healing [4]. Mesenchymal stromal cells (MSCs) may represent a good candidate cell source because of their biological characteristics and potential role in the therapy of degenerative diseases [5], [6]. MSCs are indeed easy to isolate from bone marrow and other adult tissues (*e.g.*, adipose tissue, dental pulp) [7], [8] and from prenatal tissues (*e.g.*, placenta, umbilical cord blood) [9]. MSCs are fibroblast-like cells, capable of self-renewal and in vitro differentiation toward several mesenchymal lineages including bone, fat, cartilage, tendon and muscle [10], [11]. The therapeutic effects of MSCs have been attributed both to their regenerative and trophic properties, the latter occurring through the production of bioactive products [12].

A variety of preclinical animal studies along with a series of clinical case reports have demonstrated the effectiveness of using MSCs in the treatment of orthopedic defects. Hydroxyapatite-based matrices loaded with MSCs were employed to repair segmental bone defects of critical size in animal models [13], [14]. Quarto *et al.* have reported the repair of massive human bone defects via a porous ceramic scaffold loaded with in vitro expanded bone marrow MSCs [15]. MSCs were also transplanted to enhance bone formation in spinal fusion surgery [16], [17], as well as in tendon and in ligament injuries [18]–[21].

Several scaffolding strategies have been developed to administrate MSCs in a lesion site. The main challenge is to concentrate the cells in the damaged location with an easy surgical manipulation to make them act as a therapeutic agent. To this purpose, hydrogels represent a class of scaffolds that offer several advantages, such as high water content and ability to entrap cells during their polymerization [22]. Compared to other classes of hydrogels, fibrin gel shows unique features which render it an interesting candidate for a variety of tissue engineering applications [23]. Specifically, (*i*) fibrin gel can be produced from the patient's own blood (*i.e.*, autologous scaffold), perfectly accomplishing both biocompatibility and biosafety issues [24]; (*ii*) fibrinogen (the monomer of fibrin) can be chemically cross-linked with cytocompatible initiators (*e.g.*, thrombin, calcium chloride), resulting in high post-polymerization survival of the cells entrapped during gel formation; and (*iii*) the obtained gel shows a sufficiently durable structure allowing uniform spatial distribution of cells [25], [26]. While fibrin hydrogels can naturally match the mechanic properties of many soft tissues, in orthopedic applications they are usually combined with other scaffold materials in order to obtain constructs with desired mechanical strength [27]. Fibrin-based biomaterials seem to be extremely useful in orthopedic and plastic surgery and in all those cases in which adhesiveness, scaffold resorption and efficient transport of nutrients and waste are required [28].

In previous in vitro studies, we developed and characterized fully autologous tissue engineered constructs using human MSCs, isolated from the bone marrow and embedded in autologous fibrin clots as scaffolds. After minimal *ex vivo* expansion and short-term osteogenic differentiation, MSCs were viable and retained the ability to proliferate and terminally differentiate into osteoblasts [29], [30]. In our hands, the fibrin clot proved to be an ideal support for cell delivery, confirming that this scaffold can promote cell attachment, growth and differentiation, thus representing an appealing natural biopolymer for regenerative medicine [31], [32]. Furthermore, we exposed MSCs to a limited differentiation process to preserve cell viability and function, as demonstrated later [33]. This short-term osteo-induction consists in treating cell cultures (at passage 1) with osteogenic medium for 4 days before construct preparation. This procedure makes it possible to obtain a viable and proliferating osteogenic cell population, capable of producing trophic factors, potentially useful both for self-maintenance and for the recruitment of local MSCs.

Here we report the clinical outcomes of MSC/fibrin clot constructs implanted in the atrophic pseudarthrosis of the upper limb. To assess the effectiveness and safety of our procedure, healing was monitored at 6.7 months (range 3.5–12.0 months) and clinical evolution re-evaluated at 76.0 months (range 60.0–92.0 months). Our study indicates that highly autologous treatment can be effective and safe in the long-term healing of bone non-unions. This tissue engineering approach resulted in successful clinical and functional outcomes for all patients.

## Materials and Methods

### Ethics statement

Some compassionate cases were treated in our hospital from 2005 to 2007. Patients were informed about the procedure and signed a written consent according to the Declaration of Helsinki currently in force. This retrospective study was approved by our Ethics Committee (Comitato Etico Sperimentazione Farmaco CESF, Azienda Ospedaliero-Universitaria Pisana, Pisa, Italy; file number 3766/2012).

### Patient recruitment

In the years 2005–2007, before the introduction of the new national and international regulatory laws on cell manipulation for clinical purposes, we performed a limited number of compassionate therapies in severely ill orthopedic patients. Specifically, 4 females and 4 males (mean age 44 years; range 18–73 years), affected by atrophic pseudarthrosis, were selected for upper limb revision surgery using autologous MSC/fibrin scaffold constructs. The patients had undergone one or more surgical interventions with unsatisfactory outcomes and no alternative therapy was available (Table 1). The Müller AO classification was used to sort out fracture types (Table 1) [34].

**Table 1:** Pre-intervention history of enrolled patients with final atrophic pseudarthrosis.

Patient (gender and age)	Fracture type (Müller AO Classification)	Number of previous surgeries failed
F. 45	12-A1	2
M. 27	12-B3	1
F. 73	12-C1	2
M. 61	21-B1	1
M. 51	21-B1	1
M. 46	22-A1	3
F. 18	22-A2	2
F. 31	22-C3*	3

*This fracture type involves 2 bones (ulna and radius).

### Isolation, expansion, and osteogenic pre-induction of MSCs

Bone marrow aspirates (60 ml) were obtained from both the iliac crests of the patients under local anesthesia. Plasmas and sera for clot preparation and MSC culture were obtained from autologous peripheral blood. The samples were collected at the Hematology Division of Santa Chiara Hospital, Pisa. The MSC cultures were established as previously described [29], with some modifications to ensure their suitability and safety for clinical purposes. Specifically, cells were cultured in autologous serum (6–8%, depending on the yield of the individual blood samples) and the medium was half renewed every 2–3 days until 70–80% confluence was reached (passage 1). Culture times ranged from 10 to18 days. The cells were then detached using animal free protease TrypLE Select (Invitrogen) and plated at 3,000–5,000 cell/cm^2^ for further expansion. Before cell harvesting (passage 1, 80% confluency), the MSCs were cultured with osteogenic medium for 4 days (two medium changes at 24 h and 96 h), administrating 50 µg/ml of ascorbic acid (Vitamin C, Roche, Indianapolis, IN) and 10^−6^ M hydrocortisone (Flebocortid Richter, Aventis Pharma, Milan, Italy). Medical drugs instead of standard in vitro osteogenic inducers were chosen for their safety in clinical procedures. Finally, aliquots of cells, including untreated controls, were used to evaluate osteogenic markers.

### Cytofluorimetric evaluation

Aliquots of MSCs from passages 0 and 1 (*n* = 3 patients) were incubated with CD 105 FITC-conjugated, CD 90 PE-Cy5-conjugated, anti-HLA-DR PE-conjugated, CD 34 PE-conjugated, and CD 45 PerCP-conjugated antibodies (Becton Dickinson, San Jose, CA) for 30 min at 4°C, following the manufacturer's recommendations. After incubation, the cells were washed twice and used for cytofluorimetric evaluation. Specifically, 30,000 events were acquired by FACScan cytometer (Becton Dickinson) and analyzed by CellQuest analysis software (Becton Dickinson).

### Preparation of MSC/fibrin clot constructs

The day before surgery, peripheral blood samples were collected from the patients to obtain autologous plasmas. On the day of intervention, pre-induced MSCs were harvested from tissue culture flasks, viable cells were counted using Trypan Blue solution and resuspended in 2 ml of autologous plasma (0.5•10^6^–2.0•10^6^ viable cells/2 ml plasma per tube) using 50 ml tubes (Corning) to ease further manipulation. To each tube 800 µl of CaCl_2_ were added (Bioindustria Farmaceutici, Rome, Italy) at 7 mM final concentration and were incubated for 15–30 min at 37°C to obtain MSC/fibrin clot constructs. These were immediately implanted at the site of the lesion with no further osteinductive treatment. A hemocytometer was used to count the residual cells that had remained in the clot supernatants after crosslinking. Seeding efficiency was thus evaluated as difference with respect to the seeded number. Media from cell cultures (*n* = 8 patients) were collected and analyzed at each passage to exclude viral, mycotic and bacterial contaminations. Separate sets of samples, when available, were prepared in parallel to be used for construct characterization, including viability and osteogenicity.

### Viability of MSC/fibrin clot constructs

Hematoxylin and Eosin (H&E, Sigma) staining was performed on paraffin-embedded MSC/fibrin clot sections to evaluate cell morphology. MSC viability inside the clots was evaluated with alamarBlue® (AB; Invitrogen). An AB test was performed on 2 independent sets of samples (*n* = 5 patients) at 1 h and 20 h after clot crosslinking. Briefly, after removing the exceeding fluid, 10% v/v of AB/complete medium was added and reactions were stopped after 6 h. Supernatants at 100 µl/well were photometrically assayed at 570 nm and 600 nm to calculate the percentage of reduced AB (%ABred) following the manufacturer's recommendations.

### Evaluation of osteogenic markers in MSCs and MSC/fibrin clot constructs

Alkaline phosphatase (ALP) and calcium deposition were evaluated by cytochemistry on MSCs (*n* = 8 patients) and MSC/fibrin clot constructs (*n* = 3 patients).

ALP was detected with kit #86 (Sigma), following the manufacturer's recommendations. MSCs at the second confluence, both pre-induced samples and untreated controls, were cytocentrifuged, while constructs were embedded in OCT (Bio-optica, Milan, Italy), stored at −70°C and cryosectioned. The samples were then fixed, washed and treated with Naphtol AS-BI phosphate as a substrate. The cells were counterstained with neutral red. The intracellular ALP enzyme activity was highlighted in blue.

Calcium deposition was evaluated by von Kossa staining [30]. Untreated MSCs were osteoinduced for 3 weeks using a standard in vitro procedure to assess the potential terminal osteo-differentiation of the cells [29]. Mineral deposition appeared in the form of black granules under optical light microscopy.

### Surgical technique and patients' follow-up

One or more units of constructs were prepared for each patient, depending on the extent of the lesions and considering a ratio of about 1 clot per cm^3^ of the pre-intervention lesion (Table 2). Constructs immersed in their native solution were carried to the operatory room and were implanted within 2 h from preparation. The surgical procedure involved the removal of the fixation device, which had been inserted in the previous failed interventions.

**Table 2:** Details of MSC/fibrin clot construct implant and of post-implant healing.

Patient	Number of interventions	Number of constructs implanted	Bone substitute	Time of radiographic healing (months)
F. 45	1	2	Autologous bone graft from iliac crest	5.0
M. 27	1	5	Banked homologous bone and allomatrix	3.5
F. 73	1	6	Autologous bone graft from iliac crest, synthetic bone chips	5.0
M. 61	1	4	Homologous bone chips (Osteotech)	10.0
M. 51	1	2	Synthetic bone chips	7.5
M. 46	1	5	Autologous bone graft from iliac crest	6.0
F. 18	1	4	Autologous bone graft from iliac crest	5.0
F. 31	1*	2+2 (ulna+radius)	Banked homologous bone	6.0
	1**	1+4 (ulna+radius)	Autologous bone graft from iliac crest	6.0

*A partial healing of the ulna was observed after the first intervention, while the radius still presented athrophic pseudarthrosis. **Complete healing of the ulna and the radius) occurred only after the second intervention.

Sites of non-union were completely excided and the medullary canal was opened. The bone edges were cleaned and revitalized by drilling. The constructs were thus inserted in the site of non-union and bone gaps were filled with autologous (4 interventions), homologous (3 interventions), synthetic (1 intervention) or autologous+synthetic (1 intervention) material (Table 2). Bone synthesis was then performed with plate and screws. After surgery, the patients were immobilized with plaster casts or braces for 30 days. Clinical and radiographic healing was evaluated 1 month after the intervention, and follow-ups were performed at least bi-monthly until healing was diagnosed. At longer times, radiographic analyses were conducted to monitor the efficacy and safety of this *ex vivo* procedure. Averages of short- and long-term follow-ups were 6.7 and 76.0 months respectively. Clinical follow-ups focused on the functionality evaluation of the involved joints. Electromyographic and electroneurographic analyses were pursued when necessary. Radiographic evaluation was performed via x-ray analysis.

### Statistical analysis

The results were reported as means ± standard deviations. Statistically significant differences in the ALP expression of MSCs (controls versus pre-induced cells) and in MSC/fibrin clot construct viability (1 h versus 20 h) were evaluated using the Mann-Whitney test.

## Results

### Characterization of MSCs

**Figure 1:**
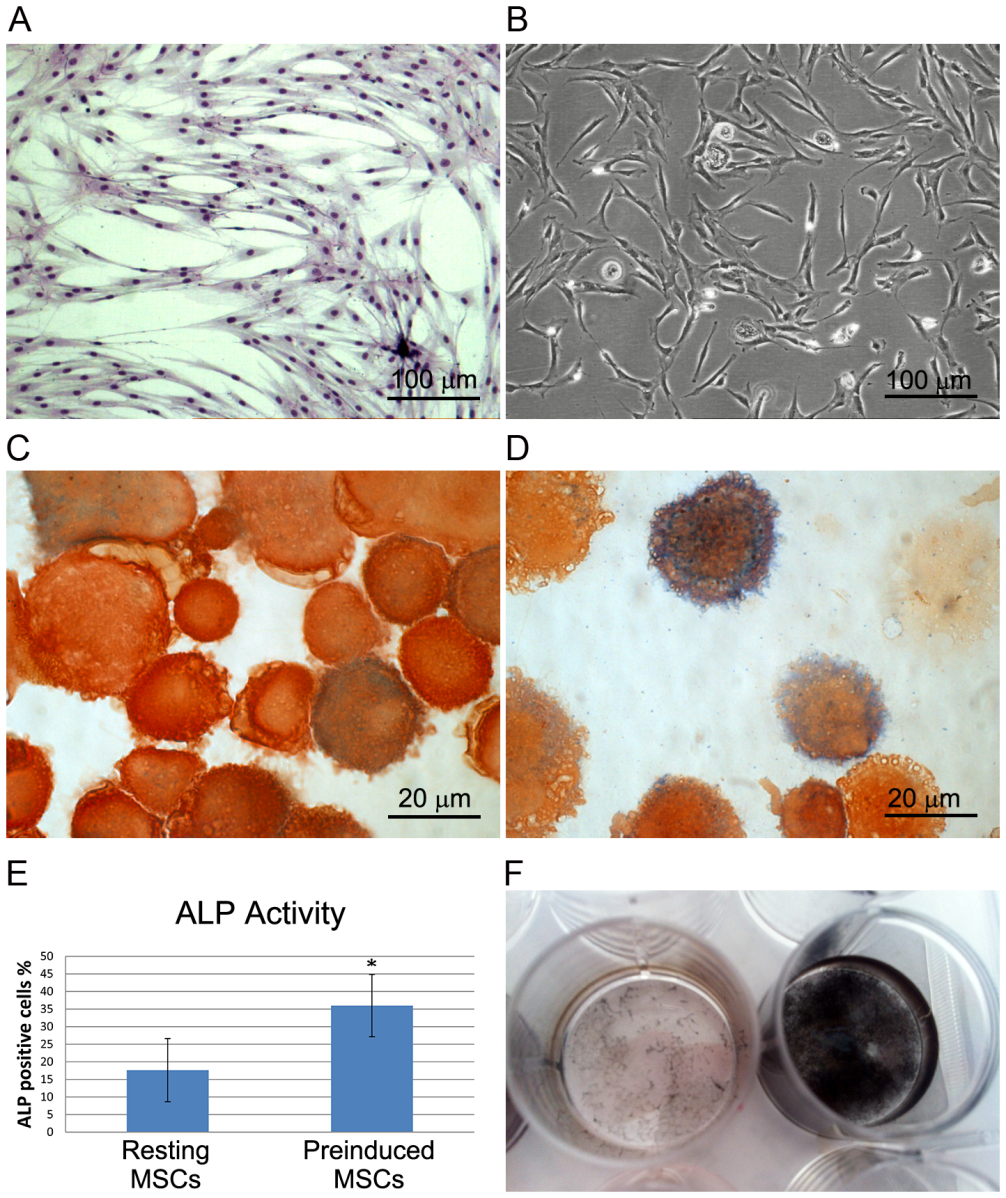
Characterization of expanded MSCs before fibrin clotting. (A) Light microscopy image of an MSC colony. (B) Light microscopy image of MSCs and MPCs in culture. (C and D) Micrographs of cytochemical staining for ALP activity (in blue) in resting and predifferentiated MSCs, respectively. (E) Bar graph showing the percentage of ALP positive cells: resting versus predifferentiated. (F) Photograph displaying von Kossa staining on untreated MSCs, detached at second confluence, and MSCs osteo-induced for 3 weeks.

Spindle-shaped cells able to form colonies, namely MSCs, were obtained in our cultures (Fig. 1A–B). Flow cytometry of MSCs at passage 0 and passage 1 showed CD105 bright, being 98.9% and 99.9%, respectively; and CD90 bright, being 97.7% and 99.0%, respectively. HLA-DR expression in both passages ranged from negative to very low fluorescence (<1%). The cells turned out to be negative for CD34 and CD45. Cell viability measured with Trypan Blue staining was 93–95%. The ALP activity of cytocentrifuged MSCs was revealed via cytochemistry (Fig. 1C–D). A quantitative analysis highlighted ALP positivity in 17.62±8.99% of untreated cells and in 36.00±8.84% of pre-induced cells with statistical significance (*p* = 0.002) (Fig. 1E). Untreated MSCs were also able to differentiate successfully into osteoblasts, as shown by calcification after 3 weeks in osteogenic medium (Fig. 1F). The supernatants from both first and second confluence turned out to be negative for the microbial detection.

### Characterization of MSC/fibrin clot constructs

MSC/fibrin clot constructs, prepared adding pre-induced MSCs to autologous plasma and calcium chloride, cross-linked in 15–20 min. This operation gave rise to jelly discs immersed in their supernatant solution. The product was ready to be implanted immediately after clotting.

**Figure 2:**
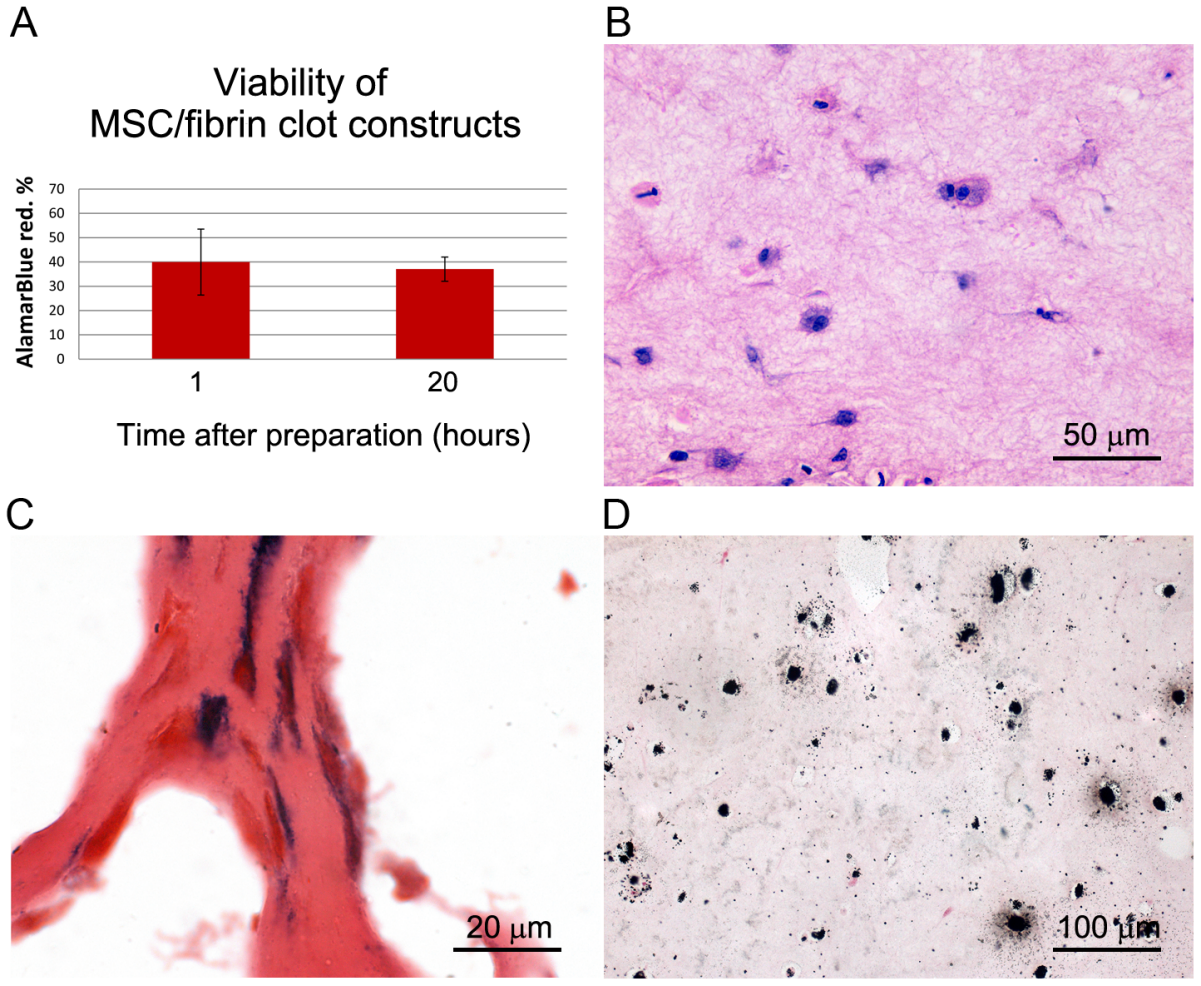
Characterization of MSC/fibrin clot constructs before implantation. MSCs inside the fibrin clots: (A) Viability of with alamarBlue assay at 1 and 20 hours after clot preparation (bar graph), (B) cell morphology with Hematoxylin and Eosin staining (micrograph), (C) ALP activity staining (micrograph). (D) Von Kossa staining (micrograph).

Separate sets of samples, prepared in parallel, were used for construct characterization. Constructs appeared stable up to 48 h after crosslinking. Cell entrapment efficiency was higher than 90%. The AB test revealed the presence of viable cells within the constructs. The analysis was carried out 1 h and 20 h after clot crosslinking and showed similar AB reduction percentages with not statistically significant difference (39.95±13.57% and 37.04±4.99%, *p* = 0.753) (Fig. 2A), demonstrating that cells can maintain their metabolism active for at least one day after clotting.

H&E stained sections showed the presence of viable MSCs embedded in the fibrin matrix, as confirmed by good cell integrity with well-defined nuclei and an ongoing mitotic process (Fig. 2B). ALP activity of pre-induced MSCs entrapped inside the clot resulted intense (Fig. 2C). Von Kossa staining on construct sections showed marked nodular calcium deposition (Fig 2D).

### Clinical evaluation

Revision of fixation at the non-union sites was performed with a short term follow-up at 6.7 months (range 3.5–12.0 months), evidencing positive clinical outcomes, with all patients recovering their limb function (Table 2). Neither peri- nor post-surgical complications were reported. Radiographic healing was detected, confirming satisfactory integration of the new bone with the surrounding tissues and abundant callus formation. Only one patient (F. 31), affected by a complex fracture involving both ulna and radius needed a second surgery. Partial healing was observed after 6 months; however a second intervention was required as the radius still presented an unsolved atrophic pseudarthrosis focus and the ulna disclosed an initial but insufficient consolidation, which showed hypertrophic pseudartrosis. The second intervention was carried out because of the breakage of a metal plate at the radius level. The fixation device was intact at the ulna level and therefore it was maintained. A bone window was thus created on the neoformed callus to host new MSC/clot units. The constructs were implanted in both bones (Table 2).

**Figure 3:**
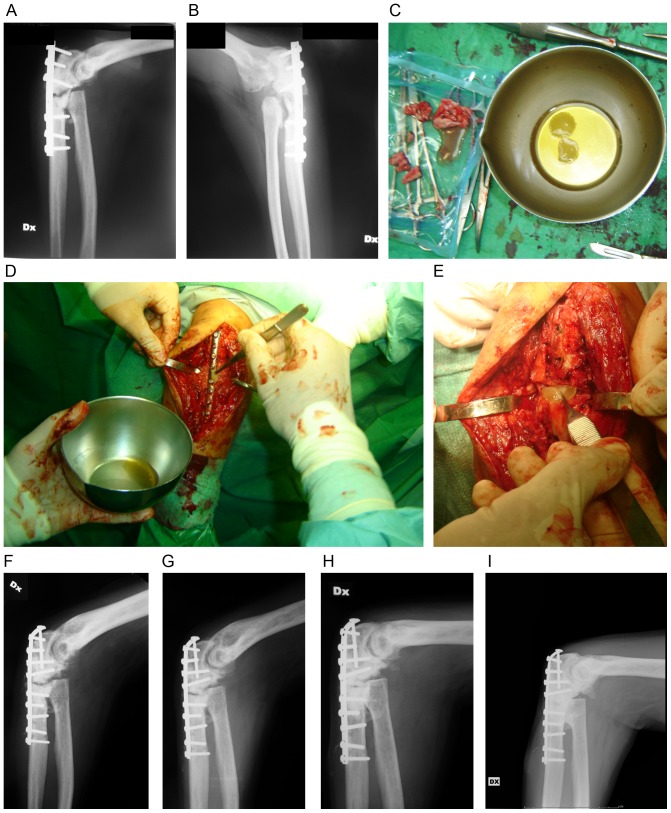
Pre- and post-implant of MSC/fibrin clot constructs in a representative patient (M.51). (A,B) Pre-intervention images showing pseudarthrosis and plate detachment. (C) MSC/fibrin clot constructs and bone chips on the operating table. (D,E) Implant of MSC/fibrin clot constructs during surgical procedure. Radiographic follow-ups at: (F) 2 months; (G) 6 months; (H) 9 months, showing occurred healing and (I) 5 years, showing bone strengthening and absence of side effects.

In all cases, subsequent long-term follow-ups at 76.0 months (range 60.0–92.0 months) showed no episodes of ectopic neoformation, infection or overgrowth, neoplastic transformation, demonstrating the safety of our procedure. Long-term efficacy was confirmed by the absence of re-fracture in all patients. A set of images relative to a representative patient (M. 51) is shown in Figure 3. Photographs provide documentary evidence of clot and bone chips on the operating table (Fig. 3C) and MSC/fibrin clot construct implanted during surgical procedure (Fig. 3D–E). Pre-intervention radiographs show presence of pseudarthrosis and plate detachment (Fig. 3A–B). Several radiographic follow-ups after the intervention with MSC/fibrin clot constructs displayed the healing timeline: after 2 and 6 months, showing initial consolidation (Fig. 3F–G); after 9 months, revealing occurred bone healing (Fig. 3H); and after 60 months, showing absence of side effects (Fig. 3I). No appreciable effectiveness differences depending on diverse bone substitute performance could be highlighted in this study.

## Discussion

Many strategies have been used in the treatment of non-unions, which are often associated with impaired healing after surgical intervention [35]. Auto-grafts, usually from the iliac crest, represent the most common therapeutic approach in trauma surgery for their osteo-induction, -production, and -conduction properties. However, several limitations, such as donor-site morbidity and supply still need to be overcome [36], [37]. Allo-grafts, on the other hand, feature good osteo-conductive properties but carry the risk of disease transmission [35]. So far, tissue engineering has appeared to be a very promising technique in several regenerative applications. Indeed, the combination of cells and scaffolds could represent the optimal solution in the management of many orthopedic defects (*e.g.*, pseudarthrosis) potentially accelerating fracture healing and reducing complications and healthcare costs [38], [39]. To manage orthopedic defects, autologous MSCs have been applied together with scaffolds at the lesion site, exploiting their trophic and differentiative properties [40]. MSC administration has been performed with two main procedures: (*i*) the “one-step” procedure, also known as “minimal manipulation”, using the bone marrow, either whole or concentrated, and (*ii*) the “two-step” procedure, also known as “extensive manipulation”, using *ex vivo* expanded and reimplanted bone marrow MSCs [41]. While the former method is simple and immediate, with an actual MSC concentration ranging in 1,000–10,000 cells/ml of bone marrow, the latter method is laborious and time-consuming, but it allows the MSC number to be increased by 100 to 10,000 folds [42].

Owing to the industriousness of the “two-step” method and to the regulatory aspects correlated to *ex vivo* manipulation, most surgeons have preferred the use of the “one-step” procedure [43]–[45]. In our vision, MSCs have to be considered as pharmaceutical agents and their concentration is fundamental for calibration with respect to defect extension. In line with this idea, there are some authors who are still encouraging the use of expanded cells [46]–[49]: Kitoh *et al.* and Kim *et al.* have used MSCs differentiated towards the osteoblast lineage, while Marcacci *et al.* and Bajada *et al.* have used undifferentiated MSCs. Cells have been employed in combination with different scaffolds: platelet rich plasma [46], macroporous bioceramic [49] and calcium sulphate pellets [48], all showing satisfactory outcomes even in difficult cases.

On the other hand, extensive *ex vivo* culture may cause abnormalities in cell morphology, such as cell inclusions, size alterations and cell detachment in the late passages [50]. Reduced cell viability due to long-term culture could hamper the efficacy of treatment. Furthermore, concerns still remain about the use of *ex vivo* expanded MSCs, including possible risks of infection and cell transformation [51], [52].

To overcome the problems related to the “two-step” procedure, we introduced modifications regarding both cell culture/induction and construct design that deserve to be discussed. Specifically, we minimally *ex vivo* expanded MSCs to reduce the risk of cell transformation in culture and we limited osteogenic induction to only 4 days. Indeed, it has been demonstrated that MSC pre-culture in osteogenic medium enhances the bone formation rate [53]. In this way, we allowed the cells to partially differentiate without reducing their proliferative potential and avoiding cell senescence. In this study, we took advantage of our previous experience in the isolation and culture of bone marrow mesenchymal cells in autologous serum for their possible clinical use. Interestingly, the autologous context made it possible to identify not only the well-known MSC population, but also a subset of cells (1–10% in mononuclear cultures), named mesodermal progenitor cells (MPCs), which appear to be precursors of MSCs [54]–[56]. These cells result to strongly adhere to plastics and therefore only a few MPCs were possibly collected at the second confluence, obtaining an almost pure MSC suspension. However, we later hypothesized that the MPC population could contribute to long-lasting healing even at a minimal concentration. Indeed, MPCs may play a significant role in the regeneration process compared to the use of mere MSCs, both for their capability to produce fresh MSCs and for their wide differentiative potential [54], [55].

The need for cell attachment, proliferation, and differentiation in tissue engineering remains crucial and requires the design of functional scaffolds, preferably bioreabsorbable. Autologous fibrin gels offer many advantages, in terms of availability, biocompatibility and biodegradation. Furthermore, a gelling scaffold can be molded to the shape of bone cavities. In previous in vitro studies, we have demonstrated that autologous plasma, cross-linked to obtain a fibrin clot, is a suitable carrier for MSCs and that the right balance between plasma and calcium chloride allows cell proliferation, terminal osteo-differentiation and spreading, showing clot-entrapped MSCs to be housed in a fibrin-microfiber porous-like structure [29]. Moreover, MSCs have been shown to exit the clot and migrating on the tissue culture plastic. This aspect suggests possible integration between the transplanted MSCs and the surrounding bone areas. During the healing process, it can be hypothesized that the MSC/fibrin clot constructs are metabolized with the same process of endogenous fibrin (*i.e.*, through fibrinolysis and phagocytosis).

In our study, we clinically demonstrate the efficacy of our construct in an autologous context. The fibrin gel texture of the clot permitted easy diffusion of the nutrients and differentiation of the inner cells towards the osteogenic lineage. The positive outcome obtained confirms the ability of MSCs in bone injury repair, encouraging tissue integration and healing. The long-term follow-up of the patients proved the safety and effectiveness of our procedures: no episodes of ectopic neoformation, infection or overgrowth, neoplastic transformation or re-fracture were detected [57], [58].

Strategies were also developed to optimize the efficiency of construct implantation during surgical treatment. In particular, we used clots immediately after their preparation to possibly allow *in vivo* osteoblast differentiation to be completed and we calibrated the number of clots to be implanted according to the extension of bone damage. Moreover, with a minimal MSC *ex vivo* expansion we managed to gather cell amounts sufficient to prepare several “units” of clots from single bone marrow samples, avoiding additional collections from patients. In our procedure, we tried to estimate an efficient ratio between extent of the lesion and MSC/fibrin clot constructs, considering them pharmacological-like agents. However, in the patient treated with a second surgery (F. 31), this ratio was doubled, since the first intervention had not been completely successful. Some *in vitro* and animal studies have reported on different performances of diverse MSC/bone substitutes, highlighting the superior seeding efficiency, metabolism and growth of MSCs on processed human cancellous allograft, as well as the best MSC proliferation and stemness maintenance on bone particles and calcium polyphosphate, with respect to some synthetic substitutes, such as hydroxylapatite and tricalcium phosphate [59], [60]. Basing on the limited number of cases treated with diverse bone fillers, differences of performance related to the substitute material could not be appreciated in our study. However, in such compassionate settings, we experienced a successful recovery only when the bone substitutes were added with MSCs.

In conclusion, our preliminary results are encouraging both in terms of bone repair efficacy and patients' safety. While the surgical approach still remains first choice for the management of non-unions, our data confirm that tissue engineering using MSCs could provide a very useful tool to accelerate and complete the healing process [61]. We believe that the minimal *ex vivo* MSC expansion and short-term osteoinduction used in this study strongly contributed to reduce risks of implant overgrowth due to uncontrolled proliferation of transplanted cells. The respect of tissue geometry, the stability of healing and the absence of neoplastic transformation at such long-term follow-ups underline the feasibility and safety of this procedure within the frame of a regenerative medicine approach.

## References

[1] Niedzielski K, Synder M (2000) The treatment of pseudarthrosis using the Ilizarov method. Ortop traumatol Rehabil 2(3): 46–48. 18034140

[2] Charles M, Barr T, Clokie CM, Sándor GK (2007) Fat embolism following posterior iliac graft harvest for jaw reconstruction: managing the complications of major surgery. J Can Dent Assoc 73(1): 67–70. 17295948

[3] Soucacos PN, Dailiana Z, Beris AE, Johnson EO (2006) Vascularised bone grafts for the management of non-union. Injury 37(1): : S41–50. Epub 2006 Apr 11. 10.1016/j.injury.2006.02.040 16581074

[4] Bruder SP, Kraus KH, Goldberg VM, Kadiyala S (1998) The effects of implant loaded with autologous mesenchymal stem cells on the healing of canine segmental bone defects. J Bone Joint Surg Am 80(7): 985–96. 9698003 10.2106/00004623-199807000-00007

[5] Horwitz EM, Le Blanc K, Dominici M, Mueller I, Slaper-Cortenbach I, et al (2005) Clarification of the nomenclature for MSC: the International Society for Cellular Therapy position statement. Cytotherapy 7: 393–395. 16236628 10.1080/14653240500319234

[6] Kassem M, Abdallah BM (2008) Human bone marrow-derived mesenchymal stem cells: biological characteristics and potential role in therapy of degenerative diseases. Cell Tissue Res 331: 157–163. 17896115 10.1007/s00441-007-0509-0

[7] Zuk PA, Zhu M, Mizuno H, Huang J, Futrell JW, et al (2001) Multilineage cells from human adipose tissue: implications for cell-based therapies. Tissue Eng 7: 211–228. 11304456 10.1089/107632701300062859

[8] Gronthos S, Brahim J, Li W, Fisher LW, Cherman N, et al (2002) Stem Cell Properties of Human Dental Pulp Stem Cells. J of Dental Res 81(8): 531–35. 10.1177/154405910208100806 12147742

[9] Barachini S, Trombi L, Danti S, D'Alessandro D, Battolla B, et al (2009) Morpho-functional characterization of human mesenchymal stem cells from umbilical cord blood for potential uses in regenerative medicine. Stem Cells Dev 18(2): 293–305. 18444788 10.1089/scd.2008.0017

[10] Pittenger MF, Mackay AM, Beck SC, Jaiswal RK, Douglas R, et al (1999) Multilineage potential of adult human mesenchymal stem cells. Science 284: 143–47. 10102814 10.1126/science.284.5411.143

[11] Minguell JJ, Erices A, Conget P (2001) Mesenchimal stem cells. Exp Biol Med 226(6): : 507–20. Review. 10.1177/153537020122600603 11395921

[12] Bonfield TL, Nolan Koloze MT, Lennon DP, Caplan AI (2010) Defining human mesenchymal stem cell efficacy in vivo. J Inflamm (Lond) 25(7): 51. 10.1186/1476-9255-7-51 PMC2987779 20974000

[13] Kon E, Muraglia A, Corsi A, Bianco P, Marcacci M, et al (2000) Autologous bone marrow stromal cells loaded onto porous hydroxiapatite ceramic accelerate bone repair in critical-size defects of sheep long bones. J. Biomed Mater Res 49(3): 328–37. 10602065 10.1002/(sici)1097-4636(20000305)49:3<328::aid-jbm5>3.0.co;2-q

[14] Arinzeh TL, Peter SJ, Archambault MP, Van Den Bos C, Gordon S, et al (2003) Allogeneic mesenchymal stem cells regenerate bone in a critical-sized canine segmental defect. J. Bone Joint Surg Am 85-A(10): 1927–35. 14563800 10.2106/00004623-200310000-00010

[15] Quarto R, Mastrogiacomo M, Cancedda R, Kutepov SM, Mukhachev V, et al (2001) Repair of large bone defects with the use of autologous bone marrow stromal cells. N Engl J Med 344(5): 385–86. 11195802 10.1056/NEJM200102013440516

[16] Muschler GF, Nitto H, Matsukura Y, Boehm C, Valdevit A, et al (2003) Spine Fusion Using Cell Matrix Composites Enriched in Bone Marrow-Derived Cells. Clin Orthop Relat Res (407): 102–18. 10.1097/00003086-200302000-00018 PMC1425047 12567137

[17] Pneumaticos SG, Triantafyllopoulos GK, Chatziioannou S, Basdra EK, Papavassiliou AG (2011) Biomolecular strategies of bone augmentation in spinal surgery. Trends Mol Med 17(4): : 215–22. Review. 10.1016/j.molmed.2010.12.002 21195666

[18] Frank C, Shrive N, Hiraoka H Nakamura N, Kaneda Y, et al (1999) Optimization of the biology of soft tissue repair. J Sci Med Sport 2(3): 190–210. 10668758 10.1016/s1440-2440(99)80173-x

[19] Shearn JT, Juncosa-Melvin N, Boivin GP, Galloway MT, Goodwin W, et al (2007) Mechanical stimulation of tendon tissue engineered constructs: effects on construct stiffness, repair biomechanics, and their correlation. J Biomech Eng 129(6): 848–54. 18067388 10.1115/1.2800769

[20] Butler DL, Gooch C, Kinneberg KR, Boivin GP, Galloway MT, et al (2010) The use of mesenchymal stem cells in collagen-based scaffolds for tissue-engineered repair of tendons. Nat Protoc 5(5): 849–63. 20431531 10.1038/nprot.2010.14

[21] Pacini S, Spinabella S, Trombi L, Fazzi R, Galimberti S, et al (2007) Suspension of Bone Marrow-Derived Undifferentiated Mesenchymal Stromal Cells for repair of Superficial Digital Flexor Tendon in Race Horses. Tissue Eng 13(12): 2949–55. 17919069 10.1089/ten.2007.0108

[22] Slaughter BV, Khurshid SS, Fisher Oz, Khademhosseini A, Peppas NA (2009) Hydrogels in Regenerative Medicine. Adv Mater 21: 3307–29. 20882499 10.1002/adma.200802106 PMC4494665

[23] Ahmed TAE, Dare EV, Hincke M (2008) Fibrin: A versatile scaffold for tissue engineering applications. Tissue Eng B 14(2): 199–215. 10.1089/ten.teb.2007.0435 18544016

[24] Jockenhoevel S, Zund G, Hoerstrup SP, Chalabi K, Sachweh JS, et al (2001) Fibrin gel—advantages of a new scaffold in cardiovascular tissue engineering. Eur J Cardiothorac Surg 19(4): 424–30. 11306307 10.1016/s1010-7940(01)00624-8

[25] Swartz DD, Russell JA, Andreadis ST (2005) Engineering of fibrin-based functional and implantable small-diameter blood vessels. Am J Physiol Heart Circ Physiol 288(3): H1451–60. 15486037 10.1152/ajpheart.00479.2004

[26] Schense JC, Hubbell JA (1999) Cross-linking exogenous bifunctional peptides into fibrin gels with factor XIIIa. Bioconjug Chem 10(1): 75–81. 9893967 10.1021/bc9800769

[27] Umeda H, Kanemaru S-I, Yamashita M, Kishimoto M, Tamura Y, et al (2007) Bone regeneration of canine skull using bone marrow derived stromal cells and beta-tricalcium phosphate. Laryngoscope 117(6): 997–1003. 17460579 10.1097/MLG.0b013e3180471459

[28] Silberstein LE, Williams LJ, Hughlett MA, Magee DA, Weisman RA (1988) An autologous fibrinogen-based adhesive for use in otologic surgery. Transfusion 28(4): 319–21. 3388476 10.1046/j.1537-2995.1988.28488265257.x

[29] Trombi L, Mattii L, Pacini S, D'Alessandro D, Battolla B, et al (2008) Human autologous plasma-derived clot as a biological scaffold for mesenchymal stem cells in treatment of orthopedic healing. J Orthop Res 26(2): 176–83. 17868116 10.1002/jor.20490

[30] Trombi L, D'Alessandro D, Pacini S, Fiorentino B, Scarpellini M, et al (2008) Good manufacturing practice-grade fibrin gel is useful as a scaffold for human mesenchymal stromal cells and supports in vitro osteogenic differentiation. Transfusion 48: 2246–2251. 18657082 10.1111/j.1537-2995.2008.01829.x

[31] Bensaid W, Triffit JT, Blanchat C, Oudina K, Sedel L, et al (2003) A biodegradable scaffold for mesenchymal stem cell transplantation. Biomaterials 24(14): 2497–502. 12695076 10.1016/s0142-9612(02)00618-x

[32] Wu X, Ren J, Li J (2012) Fibrin glue as the cell-delivery vehicle for mesenchymal stromal cells in regenerative medicine. Cytotherapy 14(5): : 555–62. Review. 10.3109/14653249.2011.638914 22175911

[33] Danti S, Serino LP, D'Alessandro D, Moscato S, Danti S, et al.. (2013) Growing bone tissue-engineered niches with graded osteogenicity: an in vitro method for biomimetic construct assembly. Tissue Eng C Methods. Apr 30. [Epub ahead of print]. 10.1089/ten.tec.2012.0445 PMC3833389 23537352

[34] Műller ME, Koch P, Nazarian S, Schatzker J (1990) The comprehensive classification of fractures of long bones. Berlin: Springer-Verlag. 53.

[35] Calori GM, Albisetti W, Agus A, Lori S, Tagliabue L (2007) Risks factors contributing to fracture non union. Injury 38(2): : S11–18. Review. 10.1016/s0020-1383(07)80004-0 17920412

[36] Heary RF, Schlenk RP, Sacchieri TA, Barone D, Brotea C (2002) Persistent iliac crest donor site pain: independent outcome assessment. Neurosurgery 50(3): 510–6. 11841718 10.1097/00006123-200203000-00015

[37] Schwartz CE, Martha JF, Kowalski P, Wang DA, Bode R, et al (2009) Prospective evaluation of chronic pain associated with posterior autologous iliac crest bone graft harvest and its effect on postoperative outcome. Health Qual Life Outcomes 29(7): 49. 10.1186/1477-7525-7-49 PMC2693524 19480692

[38] Kanakaris NK, Giannoudis PV (2007) The health economics of the treatment of long bone non-unions. Injury 38(2): : S77–84. Review. 10.1016/s0020-1383(07)80012-x 17920421

[39] Schmitt A, van Griensven M, Imhoff AB, Buchmann S (2007) Application of Stem Cells in Orthopedics. Stem Cells International 2012: : 1–11. Review. 10.1155/2012/394962 PMC3328166 22550505

[40] Cancedda R, Bianchi G, Derubeis A, Quarto R (2003) Cell Therapy for bone disease: a review of current status. Stem Cells 21: 610–619. 12968115 10.1634/stemcells.21-5-610

[41] Veronesi F, Giavaresi G, Tschon M, Borsari V, Nicoli Aldini N, et al (2013) Clinical use of bone marrow, bone marrow concentrate, and expanded bone marrow mesenchymal stem cells in cartilage disease. Stem Cells Dev 22(2): 181–92. 23030230 10.1089/scd.2012.0373

[42] Xiang Y, Zheng O, Jia BB, Huang GP, Xu YL, et al (2007) Ex vivo expansion and pluripotential differentiation of cryopreserved human bone marrow mesenchymal stem cells. J Zhejiang Univ Sci B 8: 136–146. 17266190 10.1631/jzus.2007.B0136 PMC1791057

[43] Vulcano E, Murena L, Cherubino P, Falvo DA, Rossi A, et al.. (2012) Treatment of severe post-traumatic bone defects with autologous stem cells loaded on allogeneic scaffolds. Surg Technol Int Sep 30;XXII. [Epub ahead of print]. 23065806

[44] Gómez-Barrena E, Rosset P, Müller I, GiordanoR, Bunu C, et al (2011) Bone regeneration: stem cell therapies and clinical studies in orthopaedics and traumatology. J Cell Mol Med 15(6): 1266–86. 21251219 10.1111/j.1582-4934.2011.01265.x PMC4373328

[45] Jäger M, Jelinek EM, Wess KM, Scharfstädt A, Jacobson M, et al (2009) Bone marrow concentrate: a novel strategy for bone defect treatment. Curr Stem Cell Res Ther 28(4): 34–43. 10.2174/157488809787169039 19149628

[46] Kitoh H, Kawasumi M, Kaneko H, Ishiguro N (2009) Differential effects of culture-expanded bone marrow cells on the regeneration of bone between the femoral and the tibial lengthenings. J Pediatr Orthop 29(6): 643–9. 19700998 10.1097/BPO.0b013e3181b2afb2

[47] Kim SJ, Shin YW, Yang KH, Kim SB, Yoo MJ, et al (2009) A multi-center, randomized, clinical study to compare the effect and safety of autologous cultured osteoblast (Ossron) injection to treat fractures. BMC Musculoskelet Disord 10: 20. 19216734 10.1186/1471-2474-10-20 PMC2656455

[48] Bajada S, Harrison PE, Ashton BA, Cassar-Pullicino VN, Ashammakhi N, et al (2007) Successful treatment of refractory tibial nonunion using calcium sulphate and bone marrow stromal cell implantation. J Bone Joint Surg Br 89(10): 1382–6. 17957083 10.1302/0301-620X.89B10.19103

[49] Marcacci M, Kon E, Moukhachev V, Lavroukov A, Kutepov S, et al (2007) Stem cells associated with macroporous bioceramics for long bone repair: 6- to 7-year outcome of a pilot clinical study. Tissue Eng 13(5): 947–55. 17484701 10.1089/ten.2006.0271

[50] Bonab MM, Alimoghaddam K, Talebian F, Ghaffari SH, Ghavamzadeh A, et al (2006) Aging of mesenchymal stem cell in vitro. BMC Cell Biology 7: 14. 16529651 10.1186/1471-2121-7-14 PMC1435883

[51] Lynch JR, Taitsman LA, Barei DP, Nork SE (2008) Femoral non union: risk factors and treatment options. J Am. Acad Orthop Surg 16: 88–97. 10.5435/00124635-200802000-00006 18252839

[52] Greco SJ, Rameshwar P (2012) Mesenchymal stem cells in drug/gene delivery: implications for cell therapy. Ther deliv 3(8): 997–1004. 22946432 10.4155/tde.12.69

[53] Castano-Izquierdo H, Alvarez-Barreto J, van den Dolder J, Jansen JA, Mikos AG, et al (2007) Pre-culture period of mesenchymal stem cells in osteogenic media influences their in vivo bone forming potential. J Biomed Mater Res A 82(1): 129–38. 17269144 10.1002/jbm.a.31082

[54] Petrini M, Pacini S, Trombi L, Fazzi R, Montali M, et al (2009) Identification and purification of mesodermal progenitor cells from human adult bone marrow. Stem Cells Dev 18(6): 857–866. 18991503 10.1089/scd.2008.0291 PMC3085824

[55] Trombi L, Pacini S, Montali M, Fazzi R, Chiellini F, et al (2009) Selective culture of mesodermal progenitor cells. Stem Cells Dev 18(8): 1227–34. 19331526 10.1089/scd.2009.0054

[56] Pacini S, Carnicelli V, Trombi L, Montali M, Fazzi R, et al (2010) Constitutive expression of pluripotency-associated genes in mesodermal progenitor cells (MPCs). PloSOne 5(3): e9861. 10.1371/journal.pone.0009861 PMC2845604 20360837

[57] Harris MT, Butler DL, Boivin GP, Florer JB, Schantz EJ, et al (2004) Mesenchymal stem cells used for rabbit tendon repair can form ectopic bone and express phosphatase activity in constructs. J Orthop Res 22(5): 998–1003. 15304271 10.1016/j.orthres.2004.02.012

[58] Montaln H, Schichor C, Lah TT (2010) Human mesenchymal stem cells and their use in cell-based therapies. Cancer 116(11): : 2519–30. Review. 10.1002/cncr.25056 20301117

[59] Seebach C, Schultheiss J, Wilhelm K, Frank J, Henrich D (2010) Comparison of six bone-graft substitutes regarding to cell seeding efficiency, metabolism and growth behaviour of human mesenchymal stem cells (MSC) in vitro. Injury 41(7): 731–8. 20233614 10.1016/j.injury.2010.02.017

[60] Siggers K, Frei H, Fernlund G, Rossi F (2010) Effect of bone graft substitute on marrow stromal cell proliferation and differentiation. J Biomed Mater Res A 94(3): 877–85. 20336765 10.1002/jbm.a.32766

[61] Giannoudis PV, Einhorn TA, Marsh D (2007) Fracture healing: the diamond concept. Injury 38(4): : S3–6. Review. 10.1016/s0020-1383(08)70003-2 18224731

