# Correction: Use of Autologous Human mesenchymal Stromal Cell/Fibrin Clot Constructs in Upper Limb Non-Unions: Long-Term Assessment

**DOI:** 10.1371/annotation/e4403abb-b80e-43c5-be74-1bdb2c899d1c

**Published:** 2013-11-12

**Authors:** Stefano Giannotti, Luisa Trombi, Vanna Bottai, Marco Ghilardi, Delfo D'Alessandro, Serena Danti, Giacomo Dell'Osso, Giulio Guido, Mario Petrini

During the drafting of this article for publication, a citation was incorrectly omitted from the manuscript. Indeed, some preliminary outcomes on long-term assessment for the cases reported in this article were shown in the short communication below:

Giannotti S, Bottai V, Ghilardi M, Dell'Osso G, Fazzi R, Trombi L, Petrini M, Guido G. (2013) Treatment of pseudoarthrosis of the upper limb using expanded mesenchymal stem cells: a pilot study. Eur Rev Med Pharmacol Sci 17:224-227.

This communication should be considered as a brief overview of some clinical results, the publication should have been cited in the PLOS ONE article.

Both articles relate to the same group of patients, thus they both include the patients' medical histories and consistent results about radiographic healing (short term). However, the PLOS ONE article reports an increased number of results (see Table1 and 2). Furthermore, in this publication, the average follow-up time was extended (76.0 months) with respect to that reported in the Eur Rev Med Pharmacol Sci article (50.3 months). The short communication in Eur Rev Med Pharmacol Sci did not report the experimental work related to cell culture and construct characterization which was reported in the PLOS ONE article.

